# The Management of Patients after Surgical Treatment of Maxillofacial Tumors

**DOI:** 10.1155/2016/4045329

**Published:** 2016-09-26

**Authors:** D. Rolski, J. Kostrzewa-Janicka, P. Zawadzki, K. Życińska, E. Mierzwińska-Nastalska

**Affiliations:** ^1^Department of Prosthodontics, Medical University of Warsaw, Warsaw, Poland; ^2^Clinic of Cranio-Maxillo-Facial and Oral Surgery and Implantology, Medical University of Warsaw, Warsaw, Poland; ^3^Department of Family Medicine, Internal and Metabolic Diseases, Medical University of Warsaw and Systemic Vasculitis Outpatient Clinic, Czerniakowski Hospital, Warsaw, Poland

## Abstract

Morphological and functional disturbances induced by postsurgical defects and loss of tissues in the stomatognathic system due to the treatment of tumors in the maxillofacial region determine the therapeutic needs of patients. The study aimed at clinical and epidemiological evaluation of patients under prosthetic treatment in order to establish the algorithm for rehabilitation. The study group was composed of the patients after midface surgery (45.74%); surgery in a lower part of the face (47.38%); mixed postoperative losses (3.44%); loss of face tissues and surgery in other locations in the head and neck region (3.44%). The supplementary treatment was applied in 69.63% of patients. Clinical and additional examinations were performed to obtain the picture of postoperative loss, its magnitude, and location to plan the strategy of prosthetic rehabilitation. The management algorithm for prosthetic rehabilitation in patients after surgical treatment of maxillofacial neoplasms was based on its division in stages. The location and magnitude of postoperative losses, as well as the implementation of supplementary treatment of the patients after treatment of maxillofacial tumors, influence the planning of prosthetic rehabilitation that plays a key role and facilitates the patients' return to their prior living situation, occupational and family lives.

## 1. Introduction

Head and neck cancers make about 10% of all malignant neoplasms diagnosed in humans. As much as 90% of neoplasms are of ectodermal origin and they are mostly squamous cell carcinomas (SCC) [1]. They may affect different anatomical structures, such as lips, oral cavity, oral pharynx, nasal and laryngeal parts of the pharynx, nasal cavity, paranasal sinuses, larynx, and salivary glands. The deformity and functional defects due to neoplastic diseases and their surgical treatment may lead to different stages of dysfunction, comprising basic vital functions like mastication, swallowing, and speaking.

Contemporary methods of treating tumors in the maxillofacial region, based on surgery and supplementary use of radiation or chemotherapy, have become more and more effective [2, 3]. They are still the most effective methods for treating squamous cell carcinoma of the head and neck (SCCHN). It should be emphasized that patients under treatment mostly benefit from a multidisciplinary team management (oncologic surgery, laryngology, maxillofacial surgery, radiotherapy, and prosthodontics). However, the applied treatment often contributes to numerous morphological and functional disturbances in the stomatognathic system, which greatly affect the rehabilitation of patients.

In the midface, the results of surgical approach are mostly due to the resection of maxillary bone, when the continuity of the dental arch and alveolar ridge is broken and parts of hard and soft palates, zygomatic bone, and bottom of eye socket, frequently with all its content (enucleation), are lost. In consequence of surgical treatment, the connection between oral, nasal, and sinus cavities is formed. Oral and nasal connection implicates the development of serious disturbances of basic functions of the stomatognathic system, affecting mastication, swallowing, and speaking. Eating is considerably impeded by the penetration of gastric contents and liquids into the nasal cavity, sinuses, and throat. This is because of the decreased elasticity and mobility of buccal and labial orbicular muscles, reduced mouth opening, disturbed sense-perception, and dryness of mucous membrane (xerostomia) [4]. On the other hand, disturbances within a lower part of the face after tumor-related surgical treatment occur in the maxillary bone, soft tissues of this region, and structures of the temporomandibular joint (TMJ). The class of deformation determines the kind and magnitude of disturbances in the occlusion and mobility of the mandible. The observed disturbances are mostly manifested after hemiresection with disarticulation, leading to speech disturbances and impaired mastication and swallowing ability. Within the oral cavity, the following resections are performed: cheek, tongue, structures of the oral cavity, and the walls of the throat. These resections are frequently responsible for the development of postoperative scars and adhesions, shallow vestibules and oral cavity base, limited movements of the tongue, and abnormally small oral orifice (microstomia). Difficulties in the formation of food bolus and its swallowing lead to an impaired ability to masticate and swallow.

In some cases, the tissue loss can be observed in the eyeball, ear, and nose. It may be even more extensive and comprise, beside maxillary and mandibular bones, zygomatic bone, tissues of the cheek, and orbital cavity. With the presence of such extensive mutilations and deformations, some serious mental disorders may occur in the patients [5].

The supplementary treatment (ionizing radiation, chemotherapy) beside its positive therapeutic effects in cancer treatment may also induce a variety of adverse side effects, as well as morphological and physiologic complications in hard and soft tissues of the head and neck region, which substantially impedes prosthetic treatment [6]. Radiotherapy is frequently responsible for mucous inflammation of the oral cavity, tongue, and throat (mucositis), bleeding, pain, or changes of taste. It may also cause inflammation and bone necrosis [6–8]. Serious disorders of salivary gland secretion (xerostomia) due to irreversible changes in glandular tissues, manifested by fibrosis and atrophies, are observed in irradiated patients. Changes in oral biocoenosis following both radiotherapy and chemotherapy (RTH and CHTH), the loss of salivary buffering capacity, and low level of immunoglobulins contribute to the development of prosthetic stomatopathy, frequently complicated by fungal infection and secondary bacterial infections. In this group of patients, fungal infections (candidiasis) are difficult to cure, taking account of their frequent recurrence and the need to intensify treatment by increasing antifungal drug doses and extending treatment time [8–10].

Morphological and functional disorders caused by surgical and supportive treatments exert an adverse effect on the masticatory organ. The role of prosthetic treatment is to use optimal procedures leading to the restoration of morphological and functional defects; therefore, the study was aimed at the clinical and epidemiological evaluation of the group of patients under the prosthetic reconstruction, depending on the class of postsurgical losses in the maxillofacial region due to neoplasms.

## 2. Material

In the years 1999–2013, the Department of Prosthodontics, Faculty of Medicine and Dentistry, Medical University, admitted 636 patients for prosthetic rehabilitation after the surgical treatment of neoplasms in the head and neck region. Of this number, 494 subjects (219 females and 275 males, aged 12–93 years, mean 58.36 ± 7.56 years) were eligible for the study. Prosthetic rehabilitation was planned for and carried out in all the patients of this group.

## 3. Methods

The clinical examination considered the cause of surgical procedure, medical diagnosis, supplementary treatment, general health state of the patient, and oral cavity complaints. The subjective examination was supplemented with hospitalisation records. On extraoral examination, the degree of facial deformation, the extent of dermal damage, the presence of astringent scars, and the degree of jaw movement were analysed. The intraoral examination included the assessment of postoperative tissue loss, its magnitude, and location. Based on the extra- and intraoral examinations, models of anatomic and/or functional impressions, analysis of panoramic X-ray images, and description of surgical procedures, the patients after surgery in the midface were divided into six classes (I–VI), according to Aramany's classification of postoperative losses in the maxilla [11, 12], while the patients after surgery in the lower part of the face were divided into three classes (A, B, C), using the Furuta classification [13].

On account of a wide variety of postsurgical deformations and losses, resulting from different locations of tumors, the patients were divided into four groups: patients after surgery in the midface (Group I); patients after surgery in the lower part of the face (Group II); patients with mixed postsurgical losses, in both the maxilla and mandible (Group III); and patients with losses of face tissues and those after surgery in other locations of the head and neck region (Group IV).

Three models of treatment, immediate, early, and delayed, were applied in prosthetic rehabilitation in patients after surgical treatment of maxillofacial neoplasms. These terms define the time lag between surgery and beginning of prosthetic rehabilitation. During the perioperative period (Stage 1) prosthetic rehabilitation was planned according to the predicted scheme of surgical procedures. This stage comprised the sanation of the oral cavity, endodontic, conservative, and periodontal treatments, taking maxillary impressions, manufacture of immediate denture in the form of a surgical obturator. The patients in whom an immediate reconstruction of the loss was planned or those in whom the range of tissues to be resected was difficult to predict were the only exception. No chance for immediate denture fabrication indicated the need to produce early restorations. They were produced up to seven days after surgery. After surgery within the lower part of the face, the patients were not provided with immediate denture because of technical problems in the fixation of possible restoration immediately after surgical removal of the mandibular bone and due to the mandibular mobility.

The patients in an early postoperative period (the first month after operation, Stage 2) were provided with temporal prosthetic restorations. During this period, indispensable corrections were made and restorations were relined with resilient materials, as well as with therapeutic materials, such as tissue conditioners. The control visits were scheduled every 5–7 days.

Stage 3 involved the preparation of the oral cavity for a long-term prosthetic treatment, which was planned and performed after achieving the complete stability of the prosthetic area (Stage 4).

The construction of prosthetic restorations was determined by the extent and location of the postoperative loss and the condition of the foundation area. Different types of prostheses (removable, complete and partial, and fixed or modified) were used, depending on individual conditions [14]. The following proprosthetic care and control examinations (Stage 5) were planned every 6 months.

## 4. Results

Based on the fact the squamous carcinoma was the most frequent neoplastic change diagnosed in the study group of patients (66.40%; 328/494), the decision was made to undertake surgical treatment (Table 1). The number of patients who had presented themselves for prosthetic treatment (Table 2), including a high percentage of those with postoperative bone defects, increased over the observation time.

Almost half (45.75%, 226/494) of the patients underwent neoplasm surgery in the midface (Group I), including maxillary bone defects in 208 (92.04%, 208/226) patients. Patients after surgery in the lower part of the face (Group II) formed a similar group (47.37%, 234/494). The majority (69.23%, 162/234) showed the loss of mandibular bone and 17 (3.44%, 17/494) patients showed mixed postsurgical losses in both the maxilla and mandible (Group III). There were only 17 (3.44%, 17/494) patients with losses of face tissues after surgery in other areas of the head and neck region (Group IV) (Table 1). The distribution of patients in the three classes of losses (A, B, and C) was similar.

The supplementary (RTH and CHTH) treatment was applied to 344 (69.63%, 344/494) patients.

All patients had prosthetic restorations made; they differed in structure, depending on the extent and location of postoperative defects. The production of the prosthetic restorations (removable partial or complete denture) was preceded by the preparation of the prosthetic area, including the conservative management of remaining teeth. Surgical obturator with a well-shaped obturator, prepared before surgery, carried out its task immediately after the patient's awaking from anaesthesia. Problems were faced during its fixation, especially in edentulous patients (circular sutures, screws), in everyday hygiene of the postoperative wound, and while fitting the shape and size of the obturator to the postoperative defect if the maxilla removal plan had to be changed during surgery. In such cases, the manufacture of a surgical obturator in the form of a prosthesis (without obturator) fulfilled essential postoperative functions of an immediate denture, holding a postoperative dressing and separating the oral cavity from the nasal cavity. In 78 (15.79%, 78/494) patients, prosthetic treatment was completed in the phase of immediate model or interim obturators and occlusal splints were used. This type of denture could be easily corrected or newly produced. The early treatment was parallel to the supportive treatment (RTH and/or CHTH). Various procedures and exercises were applied to improve functions of the stomatognathic system and mastication muscles.

The mode of long-term treatment could be applied after the stabilisation of the shape and dimension of the postoperative defect and prosthetic area. There were great differences in the possibilities of prosthetic rehabilitation between partially preserved dentition and clinical procedures in edentulous postoperative patients. The edentulous patients were provided with complete denture with an obturator (calyceal, full or empty inside), depending on the indication, integrated with the prosthetic base plate. The retention improvement of the complete denture with an obturator was possible due to implementation of implantoprosthetic methods. All types of prosthetic restorations were applicable in the case of preserved dentition. In 416 (82.21%, 416/494) patients, the multispecialist postoperative treatment was applied involving a number of medical and dental specialists. Of all the treated patients, edentulous subjects formed the largest group.

In patients after maxillary cancer resection (92.03%, 208/226), postoperative prostheses with obturators integrated with the base plate of removable partial or complete denture were used in 93.27% (194/208) of patients. In 14 (6.73%, 14/208) patients, postoperative maxillary defects (sinus-mouth connection) were enclosed with the skin-fascia-muscle flap (Table 3). Prosthetic restorations were produced after healing and stabilisation of the prosthetic area after reconstruction.

In patients after the mandibular bone resection (69.23%, 162/234), the construction of prosthetic restorations was also inextricably linked with the class of postoperative defects and the state of the prosthetic area. In this group of patients, prosthetic restorations were made after complete stabilisation of the prosthetic area, regardless of the reconstruction or its absence. Of the 162 patients, 35 (21.60%) had the mandibular bone reconstructed; patients were diagnosed with class B of the Furuta classification predominated in this group.

The quality and hygiene assessment of prosthetic restorations after completing the treatment showed the need to carry out preventive and therapeutic procedures to correct and change the relining, related not only to changes in the prosthetic foundation, but also to the frequent occurrence of prosthetic stomatopathy complicated by fungal infection. Mycotic examination was carried out and antimycotic treatment was implemented if necessary. In many cases, prosthetic restorations were exchanged for new ones or intraosseous implants were applied (not earlier than 2 years after termination of radiotherapy). Prosthetic restorations were frequently improved, for example, via transfer from complete overdenture to complete overdenture supported by intraosseous implants or transfer from removable denture with an obturator to permanent restoration after reconstruction of postoperative defect.

The implant-prosthetic treatment was applied in 25 (25/494) patients; 53 intraosseous implants were inserted, 8 into the maxillae and 45 into the mandible.

## 5. Discussion

According to the report released by the US National Cancer Institute, it is expected that in 20–30 years every third inhabitant of our globe will be afflicted with cancer, and neoplasms will become the leading cause of premature death in humans [1]. If the general incidence of neoplasms increases, the number of patients with neoplasms in the head and neck region will continue to rise. The survival rate among patients with neoplasms within the head and neck region remains stable (about 50%) despite continuous improvement of therapeutic methods [2].

In Poland, the incidence of malignant neoplasms also shows an upward trend. The death rate is going up as well [15, 16]. Over the years 1999–2013, the Department of Prosthodontics, Medical University of Warsaw, admitted 636 patients after surgery of neoplasms in the head and neck region. Of this number, 494 patients underwent prosthetic rehabilitation. The outcome of rehabilitation was assessed and presented in this paper. A threefold increase in the number of patients who have presented themselves for neoplasm treatment over ten recent years of observation should be stressed here (Table 2).

Surgical approach plays a major role in the management of head and neck neoplasms, especially in view of the extent to which they develop in the population of patients seeking medical care at outpatient oncology centers in Poland (T3-T4) [15]. Undoubtedly, surgical treatment of tumors saves the life of patients but at the same time leads to defects and loss of hard and soft tissues within the maxillofacial region. This results in serious morphological and functional disorders in the structures essential in the human body, such as the mandibular bone, jaw, nose, or orbital cavity [17, 18].

Nowadays, it is thought that surgical procedures should comprise not only resection but also reconstruction of lost tissues. Depending on the conditions and anticipated prosthetic rehabilitation, the reconstruction can be done parallel to the resection or as a separate procedure. The reconstruction should be targeted at restoring the continuity of removed tissues followed by the rehabilitation of the stomatognathic system. The reconstruction of the mandibular bone continuity is of a particular importance to the prosthetic treatment [19]. The comparative studies of the mastication in patients with and without reconstruction of the mandibular bone continuity evidently show that the function of the stomatognathic system is much better in the patients after reconstruction [20].

Early and delayed complications that occur after supplementary treatment, mostly radiotherapy, give also rise to subsequent problems. Both surgical and supplementary treatments lead to anatomical and functional disturbances in the stomatognathic system, defects of hard and soft tissues, fibrosis, scars, and atrophy. In addition, neurological symptoms manifested by disturbances in the function of sensory and motor nerves complicate the condition of patients. Side effects of frequently applied supplementary treatment limit the possibility of rehabilitation. They may also be responsible for delayed healing and reconstruction of the denture foundation in the postoperative defect area, which provides an even stronger justification for making provisional restorations at this stage.

Implantoprosthetic treatment, very helpful in postoperative patients, must be postponed due to complications after radio- and/or chemotherapy. Based on reports published in the national and international literature, as well as on our own experience, the time of delay can be estimated at two years after completing the supportive treatment, radiotherapy and radiochemotherapy, and at 6 months in case of chemotherapy [21–24]. In this group of patients, intraosseous implant procedures should be very precisely planned because of irreversible degenerative processes that occur in the bone tissue following RTH and/or CHTH. A very careful perioperative monitoring for cancer progression should also play a significant role. The prosthetic care should be more intensive and precise than in the patients free of neoplastic problems.

The management algorithm for prosthetic rehabilitation in patients after surgical treatment of maxillofacial neoplasms was based on its division in stages.

Stage 1 comprised a perioperative period. During this period, the rehabilitation of the patient's stomatognathic system was planned according to the predicted scheme of surgical procedures and an immediate denture was produced in the form of a surgical obturator or a base plate prosthesis with obturator. Stage 2 covered an early postoperative period (the first month after surgery, possible reconstruction, plastic surgery). During this period, prosthetic rehabilitation was provided via implementation of therapeutic and preventive measures, followed by temporary and early prosthetic rehabilitation; the patients were provided with provisional restorations; immediate and early restorations were fitted and indispensable corrections were introduced. Stage 3 comprised the interim period (2–6 months after surgery) of healing and reconstruction of the denture foundation, radiotherapy and continuation of therapeutic and preventive measures (conservative and periodontal treatments, hygiene procedures, and detection and treatment of fungal infections), and relining and correction of postoperative dentures. Occlusal splints and denture with guiding planes were applied to define and consolidate new occlusal conditions, as well as intermaxillary relations. The oral cavity was also prepared for basic long-term prosthetic treatment.

Stage 4 comprised the period of complete healing and stabilisation of the denture foundation. During this period, prosthetic treatment was implemented with use of permanent and movable restorations of more complex and laborious constructions. The exact prosthetic treatment started with making long-term prosthetic restorations, where the possibilities of prosthetic rehabilitation greatly differed, depending on the preserved or lost dentition. Corrections and relining of dentures continued and treatment of fungal infection was applied if necessary.

Stage 5—the follow-up period—comprising postprosthetic care and long-term treatment continued since prosthetic rehabilitation, manufacture of restorations, and their adaptation through the whole period of their use. During control examinations carried out every six months, the condition of the prosthetic foundation, the masticatory organ, and oral cavity hygiene were assessed.

A complete five-stage therapeutic scheme was implemented in patients with postoperative defects within the area of midface, especially in those with the connection between oral and nasal cavities and maxillary sinus formed during surgical treatment of maxillofacial neoplasms. In patients in whom a lower part of the face was operated, Stage 1 (perioperative period) was passed over and prosthetic rehabilitation began with Stage 2.

The reconstruction of lost tissues (skin-fascial-muscle flap, bone reconstruction) is the optimal approach to the management of patients after cancer surgery within the head and neck region [25–30]. In our study, a low percentage of prosthetic rehabilitation of patients who underwent reconstruction (6.73% of the maxilla and 21.61% of the mandible) was due to a considerably advanced disease, the extent of the neoplastic process, and the burden of supplementary treatment. In the group of patients after reconstruction, some possible complications associated with infections, radiotherapy, and recurrent neoplasm should be considered, as they may lead to resorption and implant loss [28]. Despite these complications, the reconstruction of lost tissues with autogenic graft is the first choice for more cases of reconstruction and dental implant-supported oral rehabilitation is most frequently considered [26, 30, 31]. Unfortunately, grafted bone combined with radiotherapy is defined as a negative prognostic factor of implant survival [32]. However, there are findings which indicate that the application of radiotherapy does not rule out implant survival [33], and peri-implantitis, insufficient soft and hard tissue, muscles dysfunction, and xerostomia should also be considered [31]. In view of the above, the planning of prosthetic rehabilitation and time of possible implant insertion plays a very important role. This helps to achieve a long-term therapeutic effect. In the posttreatment management of the patients, a number of factors, for example, impaired function of muscles or infections, should be taken into consideration as they exert an effect on the function of implant- and graft-based prosthetic restorations.

Morphological and functional disturbances induced by postoperative deformations and defects of tissues in the stomatognathic system, as well as supplementary treatment, impede the identification of the patients' therapeutic needs after surgery, planning of individual prosthetic treatment, application of an adequate construction of prosthetic restorations, and provision of interdisciplinary and multiphase rehabilitation. The observed classes of destruction after treatment of neoplasm within the head and neck region indicate the need to develop reconstruction and rehabilitation methods, as well as the adequate algorithm of procedures.

## Figures and Tables

**Table 1:** Baseline characteristics.

	% (*n*)	*X* ± SD
*Age*	12–93 years	58.36 ± 7.56

*Gender*		
Female	44.33 (219/494)	
Male	55.67 (275/494)	

*Postoperative loss*		
Group I	44.75 (226/495)	
Bone loss^1^	92.03 (208/226)	
Class I	41.83 (87/208)	
Class II	27.41 (57/208)	
Class III	6.73 (14/208)	
Class IV	8.17 (17/208)	
Class V	2.88 (6/208)	
Class VI	12.98 (27/208)	
Group II	47.37 (234/494)	
Bone loss^2^	69.23 (162/234)	
Class A	40.12 (65/162)	
A1	40.00 (26/65)	
A2	46.15 (30/65)	
A3	13.85 (9/65)	
Class B	27.16 (44/162)	
B1	9.09 (4/44)	
B2	75.00 (33/44)	
B3	15.91 (7/44)	
Class C	32.72 (53/162)	
C1	94.34 (50/53)	
C2	5.66 (3/53)	
C3	0.00 (0/53)	
Group III	3.44 (17/494)	
Group IV	3.44 (17/494)	

*Supplementary treatment*	69.63 (344/494)	
Radiotherapy	85.47 (294/344)	
Chemotherapy	4.65 (16/344)	
Radio- and chemotherapy	9.88 (34/344)	

*Cause of surgical treatment*		
Squamous carcinoma	66.40 (328/494)	
Ameloblastoma	3.24 (16/494)	
Tumor mixtus	2.43 (12/494)	
Adenocarcinoma	2.02 (10/494)	
Other	25.91 (128/494)	

^1^Aramany's classification; ^2^Furuta classification.

**Table 2:** Number of patients subjected to prosthetic treatment after surgical removal of tumors in the head and neck region by the years of observation.

Years of observation	Patients (*n*)	Location of tumors			
		Group I (*n*)	Group II (*n*)	Group III (*n*)	Group IV (*n*)
1999–2002	36	19	13	2	2
2003–2006	137	64	59	8	6
2007–2010	191	93	87	4	7
2011–2013	130	50	75	3	2

Total	494	226	234	17	17

**Table 3:** Characteristics of postoperative patients classified for prosthetic rehabilitation.

Postoperative bone loss	Prosthetic characteristics	% (*n*)
Group I		93.03 (208/226)
	Reconstruction of bone loss in maxilla	6.73 (14/208)
	Postoperative bone loss in maxilla	93.27 (194/208)

Group II		69.23 (162/234)
	Reconstruction of bone loss in mandible	21.61 (35/162)
	Postoperative bone loss in mandible	78.39 (127/162)

Group III		3.44 (17/494)
	Reconstruction of bone loss in maxilla & mandible	5.88 (1/17)
	Postoperative bone loss	94.12 (16/17)

## References

[1] (2010). *Cancer Trends Progress Report-2009/2010 Update*.

[2] Budach W., Hehr T., Budach V., Belka C., Dietz K. (2006). A meta-analysis of hyperfractionated and accelerated radiotherapy and combined chemotherapy and radiotherapy regimens in unresected locally advanced squamous cell carcinoma of the head and neck. *BMC Cancer*.

[3] Zackrisson B., Mercke C., Strander H., Wennerberg J., Cavallin-Ståhl E. (2003). A systematic overview of radiation therapy effects in head and neck cancer. *Acta Oncologica*.

[4] Beumer J., Curtis T., Marunick M. T. (1997). Maxillofacial rehabilitation: prosthodontic and surgical considerations. *Journal of Oral and Maxillofacial Surgery*.

[5] McDonough E. M., Boyd J. H., Varvares M. A., Maves M. D. (1996). Relationship between psychological status and compliance in a sample of patients treated for cancer of the head and neck. *Head and Neck*.

[6] Sonis S. T., Fey E. G. (2002). Oral complications of cancer therapy. *Oncology*.

[7] Rayatt S. S., Mureau M. A., Hofer S. O. (2007). Osteoradionecrosis of the mandible: etiology, prevention, diagnosis and treatment. *Indian Journal of Plastic Surgery*.

[8] Rubenstein E. B., Peterson D. E., Schubert M. (2004). Clinical practice guidelines for the prevention and treatment of cancer therapy-induced oral and gastrointestinal mucositis. *Cancer*.

[9] Szyszkowska A., Puławska M., Lewicka M., Koper J., Malicka M. (2011). Dental care of patients undergoing chemo- and radiotherapy. *Wspolczesna Onkologia*.

[10] Denis F., Garaud P., Bardet E. (2004). Final results of the 94-01 French head and neck oncology and radiotherapy group randomized trial comparing radiotherapy alone with concomitant radiochemotherapy in advanced-stage oropharynx carcinoma. *Journal of Clinical Oncology*.

[11] Aramany M. A. (1978). Basic principles of obturator design for partially edentulous patients. Part I: classification. *The Journal of Prosthetic Dentistry*.

[12] Aramany M. A. (1978). Basic principles of obturator design for partially edentulous patients. Part II: design principles. *The Journal of Prosthetic Dentistry*.

[13] Furuta I. M. (1993). The forms and classification of mandibular defects. *Dental Journal*.

[14] Oh W.-S., Roumanas E. (2006). Alternate technique for fabrication of a custom impression tray for definitive obturator construction. *Journal of Prosthetic Dentistry*.

[15] Didkowska J., Wojciechowska U., Zatoński W. (2010). *Malignant Neoplasms in Poland, 2009*.

[16] The National Database http://www.onkologia.org.pl/pl/p/7.

[17] Cordeiro P. G., Santamaria E. A. (2000). A classification system and algorithm for reconstruction of maxillectomy and midfacial defects. *Plastic & Reconstructive Surgery*.

[18] Weymuller E. A., Alsarraf R., Yueh B., Deleyiannis F. W.-B., Coltrera M. D. (2001). Analysis of the performance characteristics of the University of Washington Quality of Life instrument and its modification (UW-QOL-R). *Archives of Otolaryngology—Head and Neck Surgery*.

[19] Vinzenz K., Schaudy C. (2011). Osteoplastic surgery of the face—state of the art and future aspects. *International Journal of Stomatology & Occlusion Medicine*.

[20] Marunick M. T., Roumanas E. D. (1999). Functional criteria for mandibular implant placement post resection and reconstruction for cancer. *The Journal of Prosthetic Dentistry*.

[21] Eckert S. E., Desjardins R. P., Keller E. E., Tolman D. E. (1996). Endosseous implants in an irradiated tissue bed. *Journal of Prosthetic Dentistry*.

[22] Karr R. A., Kramer D. C., Toth B. B. (1992). Dental implants and chemotherapy complications. *The Journal of Prosthetic Dentistry*.

[23] Roumanas E. D., Nishimura R. D., Davis B. K., Beumer J. (1997). Clinical evaluation of implants retaining edentulous maxillary obturator prostheses. *Journal of Prosthetic Dentistry*.

[24] Werkmeister R., Szulczewski D., Walteros-Benz P., Joos U. (1999). Rehabilitation with dental implants of oral cancer patients. *Journal of Cranio-Maxillo-Facial Surgery*.

[25] Curtis D. A., Plesh O., Miller A. J. (1997). A comparison of masticatory function in patients with or without reconstruction of the mandible. *Head and Neck*.

[26] Garrett N., Roumanas E. D., Blackwell K. E. (2006). Efficacy of conventional and implant-supported mandibular resection prostheses: study overview and treatment outcomes. *Journal of Prosthetic Dentistry*.

[27] Smolka K., Kraehenbuehl M., Eggensperger N. (2008). Fibula free flap reconstruction of the mandible in cancer patients: evaluation of a combined surgical and prosthodontic treatment concept. *Oral Oncology*.

[28] Rana M., Warraich R., Kokemüller H. (2011). Reconstruction of mandibualr defects—clinical retrospective research over a 10-year period. *Head & Neck Oncology*.

[29] Takushima A., Harii K., Asato H., Momosawa A., Okazaki M., Nakatsuka T. (2005). Choice of osseous and osteocutaneous flaps for mandibular reconstruction. *International Journal of Clinical Oncology*.

[30] Doll C., Nack C., Raguse J.-D. (2015). Survival analysis of dental implants and implant-retained prostheses in oral cancer patients up to 20 years. *Clinical Oral Investigations*.

[31] Hessling S. A., Wehrhan F., Schmitt C. M., Weber M., Schlittenbauer T., Scheer M. (2015). Implant-based rehabilitation in oncology patients can be performed with high long-term success. *Journal of Oral and Maxillofacial Surgery*.

[32] Schiegnitz E., Al-Nawas B., Kämmerer P. W., Grötz K. A. (2014). Oral rehabilitation with dental implants in irradiated patients: a meta-analysis on implant survival. *Clinical Oral Investigations*.

[33] Shaw R. J., Sutton A. F., Cawood J. I. (2005). Oral rehabilitation after treatment for head and neck malignancy. *Head & Neck*.

